# A pilot study of an autologous tumor-derived autophagosome vaccine with docetaxel in patients with stage IV non-small cell lung cancer

**DOI:** 10.1186/s40425-017-0306-6

**Published:** 2017-12-19

**Authors:** Rachel E. Sanborn, Helen J. Ross, Sandra Aung, Anupama Acheson, Tarsem Moudgil, Sachin Puri, Traci Hilton, Brenda Fisher, Todd Coffey, Christopher Paustian, Michael Neuberger, Edwin Walker, Hong-Ming Hu, Walter J. Urba, Bernard A. Fox

**Affiliations:** 1 Robert W. Franz Cancer Research Center, Earle A. Chiles Research Institute, Providence Portland Medical Center, Portland, OR USA; 2 Mayo Clinic Arizona, Phoenix, AZ USA; 3 UbiVac, Portland, OR USA; 4 Laboratory of Molecular and Tumor Immunology, Robert W. Franz Cancer Research Center, Earle A. Chiles Research Institute, Providence Cancer Center, Portland, OR USA; 5 Immunological Monitoring Laboratory, Robert W. Franz Cancer Research Center, Earle A. Chiles Research Institute, Providence Cancer Center, Portland, OR USA; 6 Laboratory of Cancer Immunobiology, Robert W. Franz Cancer Research Center, Earle A. Chiles Research Institute, Providence Cancer Center, Portland, OR USA; 7 Department of Molecular Microbiology and Immunology; and Knight Cancer Institute, Oregon Health and Science University, Portland, OR USA; 8 Present address: Nektar Therapeutics, San Francisco, USA; 9 Present address: Department of General, Visceral and Transplantation Surgery, University of Munich, Campus Grosshadern, Munich, Germany; 10 Earle A. Chiles Research Institute, N.E. Glisan Street, 2N35, Portland, OR 97213 USA

**Keywords:** Non-small cell lung cancer, Vaccine, Pleural effusion, Immunotherapy

## Abstract

**Background:**

Tumor-derived autophagosome vaccines (DRibbles) have the potential to broaden immune response to poorly immunogenic tumors.

**Methods:**

Autologous vaccine generated from tumor cells harvested from pleural effusions was administered to patients with advanced NSCLC with the objectives of assessing safety and immune response. Four patients were vaccinated and evaluable for immune response; each received two to four doses of vaccine. Study therapy included two cycles of docetaxel 75 mg/m^2^ on days 1 and 29 to treat the tumor, release hidden antigens and produce lymphopenia. DRibbles were to be administered intradermally on days 14, 43, 57, 71, and 85, together with GM-CSF (50 μg/d x 6d, administered via SQ mini pump). Peripheral blood was tested for immune parameters at baseline and at each vaccination.

**Results:**

Three of four patients had tumor cells available for testing. Autologous tumor-specific immune response was seen in two of the three, manifested by IL-5 (1 patient after 3 doses), and IFN-γ, TNF-α, IL-5, IL-10 (after 4 doses in one patient). All 4 patients had evidence of specific antibody responses against potential tumor antigens. All patients came off study after 4 or fewer vaccine treatments due to progression of disease. No significant immune toxicities were seen during the course of the study.

**Conclusions:**

DRibble vaccine given with GM-CSF appeared safe and capable of inducing an immune response against tumor cells in this small, pilot study. There was no evidence of efficacy in this small poor-prognosis patient population, with treatment not feasible. Trial registration NCT00850785, initial registration date February 23, 2009.

**Electronic supplementary material:**

The online version of this article (10.1186/s40425-017-0306-6) contains supplementary material, which is available to authorized users.

## Background

Non-small cell lung cancer (NSCLC) is the leading cancer killer worldwide [1]. Most patients with NSCLC have advanced disease, and, despite recent advances in targeted and immune therapies, the goals of treatment are often palliative. Average survival for patients without driver mutations is still less than 2 years, and ultimately most patients will progress due to resistance to available therapies [2–4].

Failure of the immune system to recognize and destroy cancer cells may be a cause of cancer development and progression [5].Clinical trials with antibodies to the programmed cell death receptor-1 (PD-1), or its ligand, PD-L1, have demonstrated responses and improved disease control for patients with advanced and heavily-pretreated NSCLC [6], resulting in long-term survival for a small proportion of patients [7]. Although the majority of patients will not respond to checkpoint inhibition, results of these and other studies have led to FDA approval for checkpoint inhibitors for the treatment of advanced NSCLC, both in the first line setting, and after progression on platinum-based chemotherapy.

NSCLC without driver mutations is one of the most heavily mutated of human cancers [8], thus it is likely that NSCLC cells harbor neoantigens to which the host would not be tolerant, and against which the immune system could mount a strong, destructive immune response. Despite this, 50% of patients with the highest number of mutations still progress by 16 months [9]. This observation underscores the point that the tumor-bearing host may not provide an appropriate environment to prime anticancer immunity. Expression of neoantigens alone is insufficient without cross-presentation by antigen presenting cells in the appropriate cytokine milieu. In addition to mutated proteins, NSCLC can over-express a number of non-mutated proteins that can induce both humoral and cytotoxic T cell responses [10], and also have the potential to serve as tumor rejection antigens [11].

This study was designed to test a novel immunotherapy strategy using a vaccine enriched for short-lived proteins (SLiPs) and defective ribosomal products (DRiPs) derived from autologous tumor cells.

Tumor cells were cultured in vitro with the proteasome inhibitor bortezomib, which stabilizes SLiPs and DRiPs, shunting proteins into the autophagy pathway [12]. After inhibiting lysosomal degradation, SLiPs and DRiPs accumulate in autophagosomes (“DRiPs in blebs”; or DRibbles) [13]. Because SLiPs are not normally available to be cross-presented by APCs, we hypothesized that there would be less peripheral tolerance against SLiPs and DRiPs, and, it would thus be easier to induce immunity against them [12, 14]. Furthermore, because they are rapidly degraded by the proteasome, peptides derived from SLiPs and DRiPs would be picked up by class I molecules in the endoplasmic reticulum and would comprise the dominant epitopes presented by the MHC/HLA [13]. Vaccination with DRibbles in preclinical mouse models induced tumor-specific T cells and provided both protective and therapeutic anti-cancer immunity [15, 16].

Docetaxel improves survival and quality of life for patients with advanced NSCLC that have progressed after first-line therapy [17]. Docetaxel also produces lymphopenia [18, 19], which was shown to improve the therapeutic effect of vaccine plus GM-CSF, possibly by increasing exposure to tumor antigens during homeostatic recovery [20]. Granulocyte-Monocyte colony stimulating factor (GM-CSF)-gene modified tumor vaccines show augmented development of tumor-specific T cells in preclinical models [21] and demonstrated some efficacy in early clinical trials in patients with NSCLC [22]. We have previously reported the use of mini-pumps to provide continuous infusion of low-dose GM-CSF to the autologous NSCLC tumor cell vaccine site as an alternative to gene modification [23] and employed that strategy here.

## Methods

### Study design and entry criteria

**Fig. 1:**
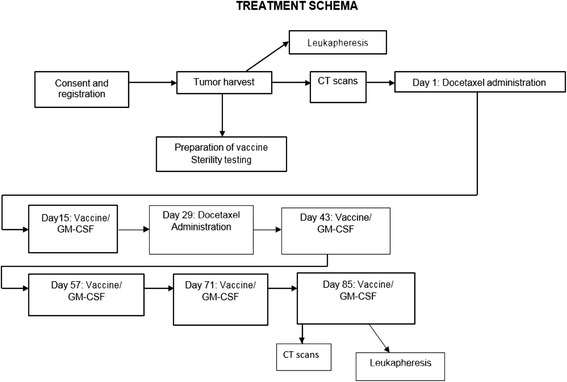
Study Schema

This single institution pilot study used autologous DRibble vaccine derived from either malignant pleural effusion or subcutaneous metastases, combined with GM-CSF infusion and docetaxel in patients with NSCLC (all patients enrolled ultimately had malignant pleural effusion as the source of vaccine). Figure 1 shows the study schema. Minimum pleural effusion volume for study eligibility was 600 cm^3^. At least 1 × 10^8^ tumor cells were needed for vaccine preparation.

Eligible patients could have received up to two prior regimens of chemotherapy for advanced NSCLC. Patients were required to have Eastern Cooperative Oncology Group (ECOG) performance status of 2 or better, be at least 18 years of age, capable of providing informed consent, and have an anticipated life expectancy of at least 6 months. Patients were required to have adequate organ function, no untreated brain metastases, spinal cord compression or significant co-morbidities or autoimmune diseases, and no prior lung cancer immunotherapy. Prior docetaxel administration was not excluded.

Patients with other active malignancies, known hypersensitivity to docetaxel, or who were HIV, hepatitis B or -C positive, were ineligible. Patients requiring chronic steroids other than as replacement for adrenal insufficiency were not eligible. Patients with deterioration of performance status beyond ECOG 2 or rapid interval progression of disease after initial study enrollment were not eligible to receive DRibble vaccine treatment. The protocol was reviewed and approved by the institutional review board at Providence Portland Medical Center, and was conducted in accordance with the Declaration of Helsinki. All patients provided written informed consent. Funding for the study was provided by the NCI, NIH R21 CA123864, and The Wayne D. Kuni and Joan E. Kuni Foundation, Vancouver, WA.

### Acquisition of human tumor cells and DRibble vaccine production

Malignant pleural effusions were collected into sterile vacuum bottles with sodium citrate as anticoagulant. The containers were transported on ice to the lab for vaccine generation, and a sample was sent to pathology for confirmation of NSCLC. Cells were harvested by centrifugation, washed in RPMI 1640 containing gentamicin, counted and resuspended in 1640 RPMI (Biowhitaker, cat# 12-702Q) containing 1.25% human albumin (ZLB Behring LLC) and 50 μg/ml gentamicin. Tumor cells were enumerated by light microscopy. When cells in excess of those required for vaccine manufacture were available, they were viably cryopreserved for use in immune function studies and attempts were made to generate tumor cell lines. DRibble vaccine was manufactured by culturing washed pleural effusion cells at 10^6^ tumor cells/ml in 30 ml media containing 100 nM of bortezomib (Velcade, Millennium Pharmaceuticals, Inc.) and 10 mM NH_4_Cl (Hospira Inc., Lake Forest, IL) in a T225 cm^2^ flask and incubated at 37 °C for 18–20 h. Culture supernatants and cells were collected as the source of DRibbles; the cells were lightly sonicated to release cell-bound DRibbles and washed with HBSS (w/o Ca2+ and Mg2+, Biowhitaker, cat# 04-315Q) at 300 x g for 10 min and the supernatant containing DRibbles transferred to a new 50 ml conical polypropylene centrifuge tube. The DRibbles in the supernatant were pelleted by centrifugation at 10,000 x g for 15 min, at 4 °C. The DRibble pellet was washed with 10 ml HBSS, making sure the pellet was resuspended thoroughly and centrifuged at 300 x g for 10 min to remove debris. The supernatant was centrifuged a second time at 10,000 x g, for 15 min, at 4 °C and the pellet resuspended in 6% Hetastarch to attain a final DRibble concentration to approximate the amount of DRibbles obtained from 5 to 20 × 10^6^ tumor cell equivalents / 500 ul. The acellular DRibble preparations were irradiated with 100Gy using a cesium irradiator (Gammacell 3000 Elan, MDS Nordion), and were aliquotted into cryovials and frozen in a -75 °C freezer. Sterility and endotoxin studies were performed on final DRibble vaccine preparations, which were stored in a monitored −80 °C freezer until use.

### Characterization of DRibble vaccines

#### Western blots

The protein concentration for each vaccine was determined using the bicinchoninic acid (BCA) assay (Pierce). Five replicates were performed for each sample. For protein analysis, 35 μl of DRibbles were mixed with 4X NuPAGE LDS sample buffer and samples were resolved by 12% SDS-PAGE (Bio-Rad). Proteins were either stained with Coomassie or transferred to a nitrocellulose membrane (iBlot, Invitrogen), incubated with primary antibodies, diluted in blocking buffer (5% NFM) overnight, and then washed and incubated with horseradish peroxidase (HRP)–conjugated secondary antibodies for 1 h. Protein bands were revealed using chemiluminescent reagents (Pierce).

#### TLR assays

HEK-Blue cells expressing a single human TLR (2,3,4,7,9) or NOD2 and a NF-κB/AP-1 inducible SEAP reporter gene (Invivogen) were used to measure TLR agonist activity in the DRibble vaccine. The Null1 cell line with the NF-κB/AP-1 inducible SEAP reporter gene (Invivogen) was used as a control. 10 μg DRibble vaccine (in triplicate) was incubated with the reporter cell lines for 16 h, after which 20 μl media was incubated with 180 μl QUANTI-Blue™ Detection media (Invivogen) for 3 h. Absorbance was measured at 600 nm on a Modulus microplate reader (Turner BioSystems). 6% Hetastarch was used as a negative control and positive controls were used for each cell line (TLR2; LTA 500 ng/mL, TLR3; poly (I:C) LMW 10 μg/mL, TLR4; LPS 500 pg/mL, TLR7; CLO97 50 μg/mL, TLR9; ODN2006 10 μg/mL, NOD2; L18-MDP 100 ng/mL).

#### ARIA-PMT flow cytometry

DRibbles were further characterized using antibodies specific for CD107a (-FITC, BD Bioscience 555,800), CD3 (-FITC, BD Bioscience 555,332), LC3 (Novus NB600–1384) and p62/SQSTM1 (Novus NBP-48320). Controls included mouse IgG_1k_-FITC (isotype control) and normal rabbit IgG (Invitrogen) as controls for murine monoclonal antibodies or rabbit antisera respectively. DRibbles were labeled with primary antibody at room temperature and placed on a continuous rotator for 30 min. DRibbles were washed with 1 ml HBSS and spun at 12,500 x g for 5 min. LC3 or p62 stained DRibble preparations were then labeled with fluorescent-conjugated antibodies and anti-rabbit-PE secondary antibody at 0.5 μg at room temperature on a continuous rotator for 30 min in the dark. DRibbles were washed with 1 ml HBSS and spun at 12,500×g for 5 min, and resuspended at 50 μg/ml in FACs buffer for analysis. Analysis of DRibbles was performed on the Becton Dickinson (BD) Aria II with an advanced FSC PMT running BD FacsDiva software.

### Leukapheresis

After confirmation of sufficient cells for vaccine generation, but prior to initiating therapy, patients underwent leukapheresis per standard Red Cross protocol to obtain cells for immune monitoring. Collections were processed by Ficoll Hypaque separation and cryopreserved in HuAB serum and DMSO. Serum was collected and stored at −80 °C. A second leukapheresis was scheduled for week 12, but all patients had progressed prior to week 12 and no post-treatment aphereses were obtained. In addition to rapid progression, the inability to obtain the week 12 apheresis severely limited the planned monitoring of T cell immune responses.

### Study therapy

Patients were treated with docetaxel 75 mg/m^2^ on day one as a one-hour infusion. Premedication (including dexamethasone) and antiemetics were administered per institutional standard protocol. Fourteen days after docetaxel, patients received an initial DRibble vaccine intradermally in the abdominal wall. The timing of DRibble vaccine administration after docetaxel administration was selected to allow for development of lymphopenia as discussed in the Background, above. The goal of the initial docetaxel administration included tumor debulking to allow time for the generation of an immune response to DRibble vaccine, and augmentation of lymphocyte depletion with the second dose in between vaccine administrations. Vital signs were obtained every 15 min after DRibble vaccine administration, and patients were observed for adverse reactions for at least one hour after the initial vaccine, and for at least 30 min after each subsequent vaccine. Vaccination sites were monitored for local reactions 48–72 h after each vaccine administration. Continuous infusion GM-CSF was administered at the vaccination site by CADD-MS 3 pump for 6 days (50 micrograms total delivered each 24 h), starting immediately after DRibble vaccination. GM-CSF was administered in this method to mimic the previous clinical models in NSCLC, with the goal of enhancing the number and function of dendritic cells at the vaccine site [23]. A second dose of docetaxel was administered on day 29, with DRibble vaccine and GM-CSF by continuous infusion given as after the first dose. A four-week regimen of docetaxel was selected in order to allow for a two-week separation from vaccine administration. Eligible patients received subsequent vaccines (accompanied by GM-CSF infusion) every 14 days, up to a total of five vaccines. Each vaccine was divided into a maximum volume of 0.5 cm^3^ for each injection, with a maximum of 7 injections per administration. The dose of vaccine for each patient was based upon the tumor cell yield.

### Assessments

Baseline imaging for tumor measurement was performed after thoracentesis. Follow up imaging for response assessment was performed between weeks 12 to 14. Toxicity was reported using the Common Terminology Criteria for Adverse Events (CTCAE) version 3.0.

#### Phenotype of tumor-associated lymphocytes in pleural effusions

Cells from pleural effusions were isolated by centrifugation and cryopreserved until samples were thawed and labeled for flow cytometry studies. The fluorochrome-labeled antibodies to CD4, CD8, CD19, CD14, CD45, and CD45RO were purchased from BD Pharmingen, CD3 and LIVE/DEAD® Fixable Yellow Dead Cell Stain Kit was purchased from Invitrogen. Stained cells were analyzed on an LSRII (BD Biosciences). Data analysis was performed using FACSDiva (Becton Dickinson) software.

#### Evaluation of tumor-specific T cells in the peripheral blood

Peripheral blood mononuclear cells (PBMC) were thawed, counted and re-suspended in X-VIVO 15 medium (Invitrogen) and plated into 24 well plates coated with anti-CD3 (10 μg/ml, Ortho OKT-3). Two days later, activated T cells were harvested, counted, re-suspended at 10^5^ cells/ml in X-VIVO15 medium containing 60 IU/ml IL-2 (Prometheus) and plated into 6 well plates for 5 to 6 days of culture in 5% CO_2_ at 37 °C. Resulting effector T cells were harvested and assayed for functional activity by measuring the release of cytokine following 18–20 h culture with autologous tumor cells. Controls included stimulation with allogeneic melanoma or NSCLC cell lines, immobilized anti-CD3 (positive control), and no stimulation (negative control). Cytokine release was assessed using commercially available cytokine ELISA or CBA kits and assays were performed according to the manufacturers’ instructions. Samples were assembled and evaluated in duplicate, and mean cytokine concentration is presented (Fig. 5).

#### Evaluation of humoral immunity by ProtoArray

Serum samples from pre- (week 0) and the latest post-treatment time point were profiled on ProtoArray® Human Protein Microarrays v5.0 (Invitrogen, Carlsbad, CA) containing approximately 9000 human proteins. One ProtoArray slide was used for each time point and slides were processed according to the manufacturer’s instructions and scanned using an Axon GenePix 4000B fluorescent microarray scanner (Molecular Devices, Sunnyvale, CA).

#### Protein microarray data analysis

Pixel intensities for protein location on the array were determined from the GenePix Pro 6.0 software (Molecular Devices) and analyzed using ProtoArray® Prospector 5.2.1 software (Invitrogen). Signal intensities were normalized across arrays by quantile normalization before fold change was determined. A minimum intensity of 1000 RFU at the post time point was required for a response.

### Statistical analysis

Due to the small number of patients in this pilot study, statistical analysis was limited to descriptive statistics. For the purposes of this study, antibody responses that were increased 5-fold or greater in the post treatment serum sample compared to baseline were considered important.

## Results

### Patient characteristics

Six patients were enrolled in the study between June 2009 and September 2011, when the study was closed due to slow accrual. Seven patients signed the informed consent; one patient was ineligible secondary to brain metastases requiring irradiation; six patients received day 1 docetaxel, and 4 patients received at least one vaccine. Median patient age was 64 years, with a range of 54 to 81 years. Three men and three women enrolled, each with ECOG performance status 1 on day 1. All patients had stage IV adenocarcinoma of the lung. The average number of prior systemic therapies was one; two patients had also received prior radiation therapy (two treated with whole brain radiation, one patient received palliative radiation to the eye). All patients had previously received platinum-based doublet chemotherapy, either with paclitaxel or pemetrexed (three with bevacizumab as well). Two patients had previously received a second line of therapy (one with pemetrexed, one with erlotinib). All patients were Caucasian. Patient characteristics are summarized in Additional file 1: Table S1.

### Characterization of the DRibble vaccine

The composition of the DRibble vaccine proteins varied among the patients. All DRibble preparations contained several putative tumor-associated antigens (Table 1). These included Eno1 [24], LDHB [25], NPM1 [26], PKM2 [27] and KPNA2 [28], which are commonly overexpressed in NSCLC, are associated with poor prognosis, and are therefore potentially relevant targets for cancer immunotherapy. ENO1 was detected in all four DRibble preparations. NPM1 was detected in three (Patients 2, 5, and 6), PKM2 was detected in two (Patients 5 and 6), while LDHB and KPNA2 were detected in one each (Patients 5 and 6, respectively). Two of these antigens (LDHB and NPM1) have recently been reported to be targets of humoral immunity and correlate with proteins for which peptides could be eluted from HLA MHC class I molecules of NSCLC cell lines [10].

**Table 1:** Characteristics of DRibble preparations

					Antigens^a^						DAMPs		
Patient ID	Tumor #	# vac given	Tumor Cell Eq/vaccine	∝g Protein/vaccine	P62	NPM1	KPNA2	LDHB	ENO1	PKM2	HSP90	HSP70	CALR
Patient 2	LT80	2	1.32E + 07	730	++	+++	ND	ND	+++	ND	+	+	+++
Patient 3	LT79	3	1.65E + 07	125	+	ND	ND	ND	+++	ND	ND	+++	+
Patient 5	LT84	4	2.10E + 07	560	+++	+	ND	+++	+	+	ND	+++	+
Patient 6	LT96	2	4.00E + 07	480	+++	+++	+	ND	+++	+++	+	+++	+++

DRibble vaccine was produced from autologous tumor and protein content was determined by the bicinchoninic acid assay (BCA). Proteins were identified by Western Blot and assigned plus symbols based on intensity of the blot (*NPM1* nucleophosmin, *KPNA2* karyopherin alpha 2, *LDHB* lactate dehydrogenase B, *ENO1* enolase 1); *PKM2* pyruvate kinase isozymes M1/M2, *HSP* Heat-shock protein, *CALR* Calreticulin. ND: Not Determined. ^a^Antigens detected by Western blot

Damage-associated molecular pattern molecules (DAMPs) are an important group of sensors, inducers and mediators of a stress response that can facilitate antigen presentation and are associated with immunogenic cell death. Heat shock protein 70 (HSP70) and calreticulin were two DAMPs found in all 4 DRibble preparations, HSP90 was detected in two (Table 1). The autophagosome-associated protein, p62, was detectable in all vaccines.

**Fig. 2:**
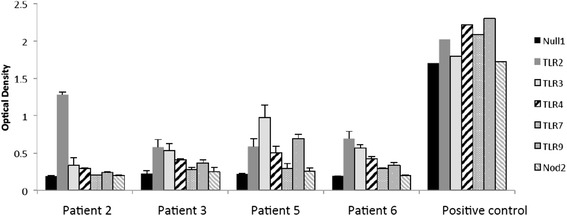
LR agonist activity of patient-derived autologous DRibbles. HEKBlue reporter cell lines (TLR2, TLR3, TLR4, TLR7, TLR9, NOD2 and control cell line Null1) were incubated with 20ìl autologous DRibbles (Patient 2, Patient 3, Patient 5, Patient 6) (in triplicate) for 16 hrs. SEAP secretion in the media was measured in Quanti-Blue media by absorbance at 600nm and compared to cell line specific positive controls

Each of the autophagosome preparations exhibited TLR agonist activity as depicted in Fig. 2. TLR2 activity was evident in all, and DRibbles generated from patients 3, 5, and 6 also expressed TLR 3 activity. DRibbles from patients 5 and 6 exhibited TLR4 agonist activity and patient 5 DRibbles also contained a functional TLR9 agonist.

**Fig. 3:**
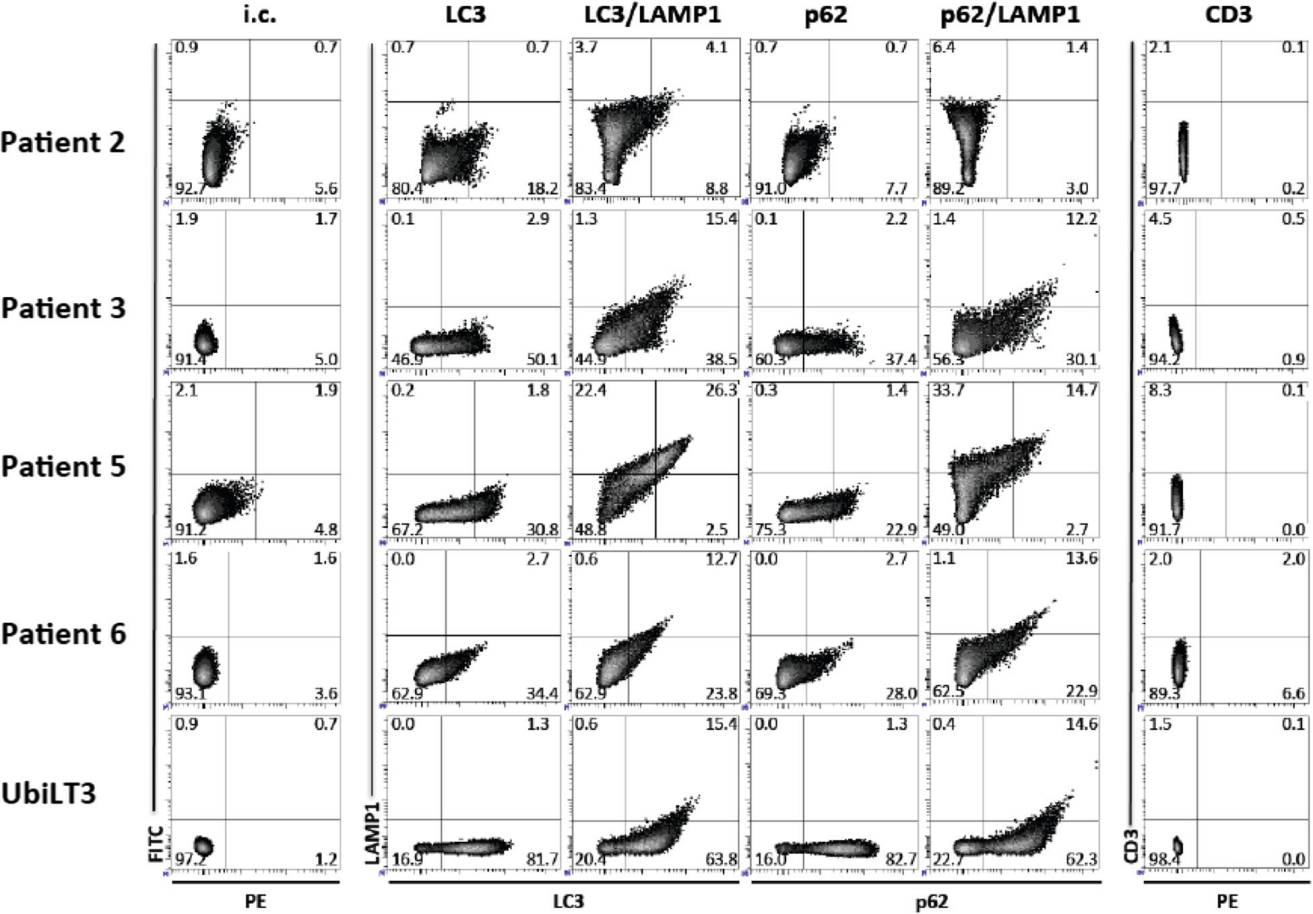
Phenotype of autologous DRibbles derived from pleural effusions compared to DRibbles from the NSCLC cell line UbiLT3. Autophagosomeenriched DRibbles for patients 2, 3, 5, 6, and UbiLT3 DRibbles, derived from a lung tumor cell line, used as a control, were labeled with autophagosome specific antibodies [anti-LC3 (PE), anti-LC3 (PE)/LAMP1-FITC, anti-p62 (PE), or anti-p62 (PE)/LAMP1-FITC]. Normal rabbit IgG (isotype control; i.c.) and CD3-PE were used as controls. Patient-derived DRibble preparations were more heterogeneous compared with cell-line derived UbiLT3

Flow cytometry was used to profile the vesicles that comprise the DRibble vaccines. Autologous DRibbles from pleural effusions were compared to DRibbles made from the UbiLT3 cell line (Fig. 3). DRibbles were labeled with antibodies specific for the autophagosome marker LC3, the autophagosome-associated protein p62, and the lysosome and autolysosome marker LAMP1. An antibody specific for CD3 was used as a control for non-specific binding. DRibbles from patient 2 were highly auto-fluorescent in the fluorescein isothiocyanate (FITC) channel with low specific LC3, p62, or LAMP1 staining. DRibbles from patients 3, 5, and 6 contained vesicles positive for LC3 (30.8–50.1%) and p62 (22.9–37.4%), showing that DRibble preparations contained autophagosomes. LAMP1 staining was highest in the DRibbles from patient 5 (22.4% LAMP1 + LC3-, 26.3% LAMP1 + LC3+, 33.7% LAMP1 + p62-, 14.7% LAMP1 + p62+), while patients 3 and 6 had more vesicles that were LC3+ and p62+, but LAMP1-. These data indicate that while the composition of autologous DRibbles can vary from sample to sample, they all contain autophagosomes and autolysosomes, the novel antigenic cargo carriers that DRibbles are posited to deliver.

### Treatment

All six eligible patients underwent baseline leukapheresis, and received day 1 docetaxel (see CONSORT diagram, Additional file 2: Figure S1). Five patients received full-dose docetaxel on day 1 (75 mg/m^2^), while one patient required initial dose reduction due to abnormal liver function tests (60 mg/m^2^). Only four patients received the first dose of DRibbles as planned on day 15. All four of those patients completed their GM-CSF infusions and received the day 29 dose of docetaxel, the day 43 DRibble vaccine, and GM-CSF. One patient required a one-week treatment delay due to pneumonia.

Two patients were able to receive the day 57 DRibble vaccine and GM-CSF. One patient received the day 71 vaccine and GM-CSF; however, no patient underwent a second leukapheresis at week 12 as initially planned. Overall, two patients did not receive vaccine, two patients received 2 vaccines each, one patient received three, and one patient received four vaccines.

Finding patients eligible for this study was difficult, and the study ultimately was halted due to slow accrual. The primary limitation was the identification of patients with malignant pleural effusions whose performance status was adequate both for study eligibility and to allow time for DRibble preparation (17 days from the time of pleural fluid collection until the completion of sterility testing after vaccine generation). Furthermore, the ability to administer DRibbles to patients serially was hampered by rapid clinical progression in this poor-prognosis population.

### Toxicity

Adverse events are shown in Tables 2 and 3. The majority were Grade 1 or 2 events (37 events; 57%), with 27 Grade 3 or 4 events reported (41.5% of total). Two Grade 5 events were reported: neutropenic fever/sepsis in a patient who did not receive a DRibble vaccine, and respiratory failure in a patient with pneumonia who received 2 doses of docetaxel and two DRibble vaccines. Adverse events were attributed primarily to docetaxel or underlying disease progression. The administration of DRibbles was well tolerated, with only one toxicity attributed to DRibble vaccine, a Grade 1 injection site reaction.

**Table 2:** Hematologic Toxicities

Toxicity	Grade 1 N (%)	Grade 2 N (%)	Grade 3 N (%)	Grade 4 N (%)	Grade 5
Anemia			1 (16.7%)		
Febrile Neutropenia/Sepsis			1 (16.7%)		1 (16.7%)
Leukopenia		1 (16.7%)	2 (33.3%)	1 (16.7%)	
Lymphopenia	3 (50%)	4 (66.7%)	3 (50%)		
Neutropenia			2 (33.3%)	2 (33.3%)	

Hematologic toxicities were attributed to docetaxel, not DRibble vaccine

**Table 3:** Non-hematologic Toxicities

Toxicity	Grade 1 N (%)	Grade 2 N (%)	Grade 3 N (%)	Grade 4 N (%)	Grade 5 N (%)
Dyspnea		2 (33.3%)	2 (33.3%)	1 (16.7%)	
Respiratory Failure				1 (16.7%)	1 (16.7%)
Upper Respiratory Infection		1 (16.7%)			
Cough		1 (16.7%)	1 (16.7%)		
Pneumonia				1 (16.7%)	
Hallucinations			1 (16.7%)		
Dehydration	1 (16.7%)		1 (16.7%)		
Hypotension		1 (16.7%)	1 (16.7%)		
Dermatology, Other (Heating pad burn)	1 (16.7%)				
Pain		1 (16.7%)	1 (16.7%)	1 (16.7%)	
Peripheral Neuropathy	1 (16.7%)				
Hyperglycemia	1 (16.7%)				
Infection		1 (16.7%)			
Sore throat	1 (16.7%)				
Flu-like symptoms	1 (16.7%)				
Fatigue	1 (16.7%)	4 (66.7%)			
Arthralgia	1 (16.7%)				
Headache	1 (16.7%)				
Fever	2 (33.3%)				
Decreased level of consciousness			1 (16.7%)		
Nausea/Vomiting	1 (16.7%)				
Hyponatremia				1 (16.7%)	
Hypoxia			1 (16.7%)		
Syncope			1 (16.7%)		
Rash	1 (16.7%)				
Injection site reaction^a^	1 (16.7%)				
Infection, Other (Lip)^b^		1 (16.7%)			
Dizziness	1 (16.7%)				
Constipation	1 (16.7%)				
Hypoalbuminemia		1 (16.7%)			

^a^Attributed to DRibble vaccine

^b^Attributed as unlikely related to DRibble vaccine

All other toxicities were attributed as unrelated to DRibble vaccine, but were considered related to docetaxel or underlying disease

Grade 3/4 hematologic toxicities (12 events; 44.4%) included leukopenia, lymphopenia, neutropenia, and anemia. One episode of grade 3 febrile neutropenia was reported, with the patient subsequently continuing on study therapy. The majority of the Grade 3/4 events were non-hematologic (55.6%; 15 events); seven events were due to pulmonary symptoms (dyspnea, hypoxia, cough, pneumonia, respiratory failure).

Two patients died during therapy, neither of whom received a DRibble vaccine; one developed altered mental status on day 11 after chemotherapy (attributed to docetaxel), had progressive functional decline, and was discharged to hospice, and the other was admitted to hospital on day 4 after docetaxel with hyponatremia, subsequently developed neutropenic fever with sepsis, and expired due to metastatic lung cancer and sepsis.

### Efficacy

No patient remained on study at the time of planned disease assessment at Day 85. At this time all six patients had discontinued therapy because of disease progression, and three had died. One patient underwent early disease assessment because of symptoms of tumor progression, and progressive disease was confirmed at Day 44. All patients are deceased. Median survival from Day 1 of treatment was five months (range 11 days-16 months). No patient received subsequent anticancer therapy.

### Characterization of the immune cells in pleural fluid

**Fig. 4:**
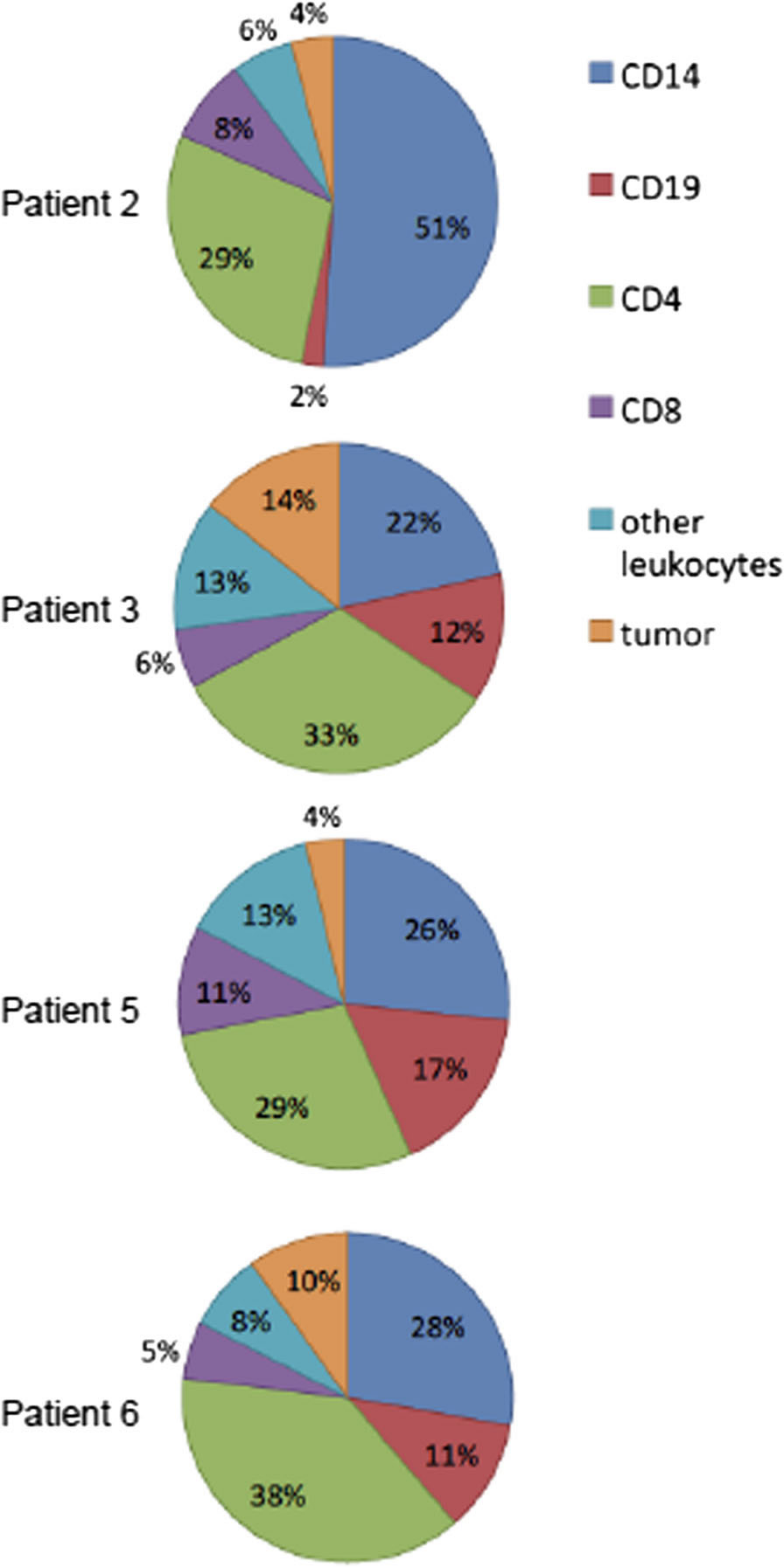
The immune cells contained in the pleural effusion were examined by flow cytometry. Phenotype was determined by staining with anti-CD45 and lineage-specific markers. Tumor cells were identified by light scatter characteristics and defined as live cells without the CD45 and lineage-specific markers. Pie charts depict percentages of live cells detected in pleural effusions for each of the patients. Phenotypes represented are CD14+ monocytes (blue), CD19+ B cells (red), CD4+ T cells (green), CD8+ T cells (purple), other leukocytes (light blue) and tumor cells (tan)

Based on light scatter properties and the absence of CD45, we estimated that tumor cells comprised from 4%–14% of viable cells in the pleural effusions (Fig. 4). Immune cells comprised the majority of cells detected in the pleural effusions, with similar profiles in 3 (patients 3, 5 and 6) of 4 patients (Fig. 4). Antigen-experienced memory and effector cells (CD45RO+) comprised the majority of CD4 (74–84%) and CD8+ (59–61%) T cells contained in the pleural fluid from patients 3, 5 and 6, while only 36% of CD8+ T cells from patient 2 were CD45RO+ (data not shown).

### T cell responses to autologous tumor cells

**Fig. 5:**
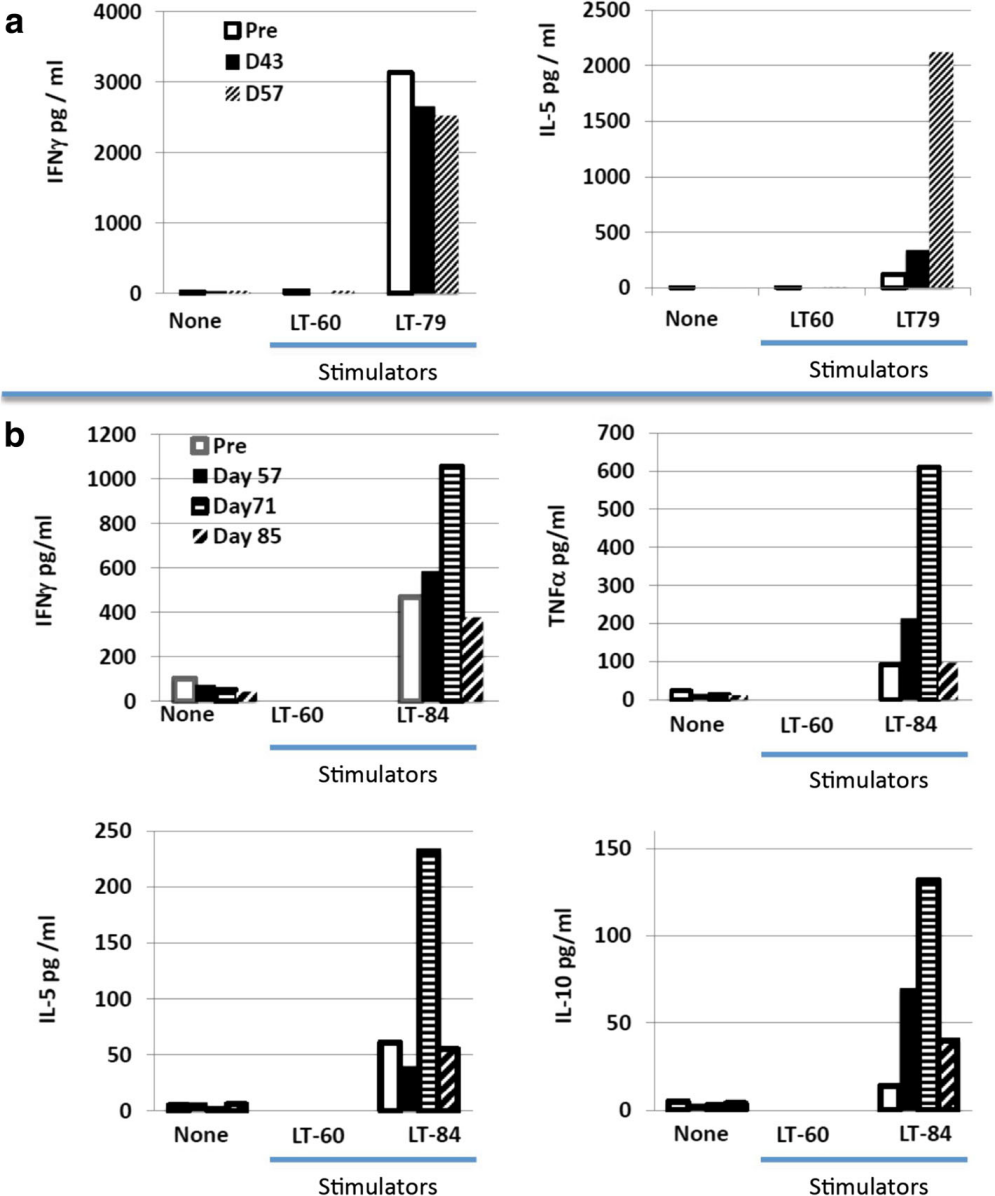
Effector T cells generated from PBMC were cultured alone or with allogeneic or autologous tumor cells and cytokine secretion was measured. PBMC from Patients 3 (**a**) and 5 (**b**) were collected at times specified in the figure legend. T cells were then cultured either alone (none), with allogeneic tumor cells (LT-60), or with the autologous tumor cells, LT-79 and LT-84 for patients 3 and 5 respectively. After 18-20 hours of culture, supernatants were collected and the concentration of cytokine, specified in the legend, were measured

Patients’ PBMCs were expanded as specified in the methods and co-cultured with autologous tumor cells (when available) or control allogeneic tumor cells in an attempt to detect tumor-specific reactivity. This was the case for tumors from patients 3, 5 and 6. For patient 2 there were only sufficient tumor cells to manufacture the vaccine. Among the three patients with autologous tumor cells available for this assay (patients 3, 5, and 6), patient 6 had PBMC only available following a single vaccine (Day 43) and no IFN-γ response was detected against his autologous tumor cells (data not shown). Of the two patients (3 and 5) who were evaluable 14 days following their second (patient 3, D57) or third and fourth vaccine (patient 5, D71 and D85), both developed new or augmented cytokine responses to autologous tumor cells (Fig. 5). Patient 3, who demonstrated a strong (>3000 pg) pre-existing IFN-γ response, developed a strong (>2000 pg) tumor-specific IL-5 response. Patient 5, the only patient evaluable 14 days after their third vaccination (D71), demonstrated a vaccine-induced increase in tumor-specific secretion of IFN-γ and TNF-α that was respectively 2-fold and 6-fold higher than baseline. Interestingly, this patient also exhibited an increase over pre-existing baseline tumor-specific IL-5 and IL-10 cytokine responses (Fig. 5). The decline in tumor-specific cytokine secretion for patient 5 by day 85 coincided with the detection of tumor progression. Given that no patient underwent week 12 leukapheresis, further immune evaluation was limited.

### Evaluation of humoral immune responses

Given the difficulty of discerning which epitopes were recognized by patients’ T cells following administration of a complex autologous tumor cell-derived vaccine, we hypothesized that humoral responses would provide clues both to the breadth and specificity of the T cell response. While data are limited, this concept is supported by several authors who used humoral immune responses to identify T cell responses in patients with prostate and NSCLC [29, 10]. Serum IgG antibody responses, which require antigen-specific CD4 T cell help, were assessed before and after vaccination in day 71 sera in patients 3 and 5. Samples for patient 2 and 6 were assessed at baseline and day 43 or 45. Patients 2, 3, 5 and 6 had 16, 18, 4 and 11 increased (>5 fold) antibody responses, respectively. Patient 2 made strong 5× antibody responses to 16 proteins (ZADH2, RRAGB, PFN2, RIMS3, C7orf62, GPM6A, MED9, FRG1BP, RPS6KB1, ETV4, ABL1, ZAP70, SPRR2G, SF3A3, PIM2, and RCHY1), patient 3 responded to 18 proteins (HIC2, AKT1S1, NPM1, cDNA clone IMAGE:5,271,031, ALOXE3, MAD2L1, RPS6KB1, MKNK1, BPHL, SLC29A1, DNAJB1, SNAPAP, EIF2B2, RRAGB, LIMK1, DNAJC5, ZC3HAV1L and NDUFB6), patient 5 had strong antibody responses against 4 proteins (LRSAM1, GPM6A, PLK2 and IMPA2) and patient 6 had antibody responses against 11 proteins (SMAD3, ARHGEF16, NOC2L, SMAD2, EIF5, ATF1, C7orf28B, NME7, DTNBP1, ERBB2 and EPHB4).

**Fig. 6:**
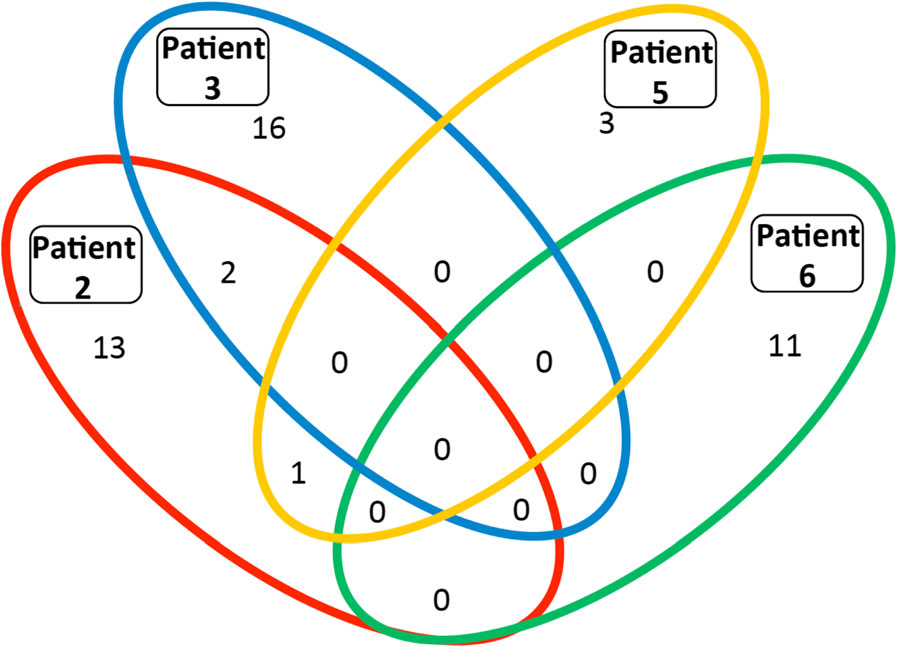
Increased antibody responses after vaccination. Serum from pre-treatment and day 71 (patients 2, 3 and 5) or day 43 (patient 6) was assessed for antibody responses against 9,000 human proteins. After data filtering, the number of antibodies with a greater than 5 -fold increase (with a minimum RFU value > 1000 post vaccination) for each patient were calculated and presented above in a Venn diagram, with shared humoral responses represented within overlapping sections

We investigated whether the strong antibody responses produced by different patients recognized any of the same proteins. Among the proteins to which strong antibody responses were made, none were recognized by antibodies in all four patients (Fig. 6). Patients 2 and 3 both made responses to ribosomal protein S6 kinase, 70 kDa, polypeptide 1 (RPS6KB1), which is overexpressed or amplified in 19% of TCGA lung adenocarcinoma samples (provisional database, as of June 2017) and 10% of squamous cell lung cancer samples (provisional database; as of June 2017). The other shared 5× responses are glycoprotein M6A (GPM6A) (patients 2 and 5) and Ras-related GTP binding B (RRAGB), transcript variant RAGBl (patients 2 and 3). Interestingly, the proteins targeted by the strong antibody responses are products of genes noted to have an amplification, mRNA upregulation or mutation in patients with adenocarcinoma of the lung (LUAD database, TCGA). This opens the possibility that these strong antibody responses and the associated CD4 T cell responses against the same protein that are required for IgG class-switching could be targeting antigens relevant for these patients. Additional support for the relevance of immune responses against these proteins is a recent report that peptides from two of the proteins targeted by an antibody response in patient 3 (NPM1 and NDUFB6) were reported to be presented by HLA class I of NSCLC cell lines [10].

## Discussion

Our group has a long-standing interest in autologous tumor cell vaccines that is grounded in the preclinical work of Prehn and Main, who documented that whole cell vaccines were most effective at providing protection from a tumor challenge with the same tumor cells comprising the vaccine [30]. Vaccines derived from different tumors of the same histology, induced by the same carcinogen, were not effective. One explanation was that unique tumor-specific rejection antigens were present in each independently-derived tumor. In our previous pilot study of autologous NSCLC vaccines genetically modified to express GM-CSF, survival was increased in the cohort of patients whose vaccines made >40 ng GM-CSF/10^6^ cells/24 h [31]. This approach was limited by the requirement for surgery in stage IV patients to obtain tissue to prepare the vaccines. In a follow-up study, autologous tumor cells were mixed with a bystander cell line (K562) engineered to secrete GM-CSF. This strategy failed to improve patient survival [22].

Immunotherapy with checkpoint blockade for lung cancer has demonstrated significant efficacy in a small proportion of patients, with checkpoint inhibitors now approved for patients with advanced NSCLC [32–35]. Although nivolumab has demonstrated unprecedented long-term survival in a few heavily-pretreated patients with NSCLC, with three-year overall survival reaching 18% in a phase I study [36], only a minority of patients experience this longer-term benefit. Long-term overall survival data is not yet available for pembrolizumab in patients with previously-untreated advanced NSCLC with >50% tumor PD-L1 expression. Although pembrolizumab is FDA-approved as a single agent in this setting, as well as in combination with carboplatin and pemetrexed for advanced non-squamous NSCLC regardless of PD-L1 status based upon superior progression-free survival and response rates compared with chemotherapy (4 month improvement in progression-free survival compared with chemotherapy), only 45% of patients with single agent, and 55% of patients receiving the triplet combination, experience disease response [37, 38]. Strategies that increase immune recognition and immune control are needed. Combination of anti-PD1 and anti-CTLA-4 inhibitors demonstrated superior response rates compared with monotherapy in advanced melanoma [39], as well as in advanced NSCLC in a few early phase trials, with ongoing cohort expansion and phase III trials for confirmation of activity pending [40, 41].

This trial, which was performed before the efficacy of checkpoint blockade had been demonstrated, investigated a novel autologous vaccine containing multiple specific tumor-associated antigens created from short-lived proteins (SLiPs) and defective ribosomal products (DRiPs) packaged in a double membrane microvesicle. We hypothesize that a major benefit of the DRibble vaccine strategy is the increased presentation of multiple overexpressed antigens that are not normally available for cross-presentation because they are short-lived [42]. This autologous DRibble vaccine, generated from cells collected from malignant pleural effusions in patients with NSCLC, generated an IgG antibody response against multiple discrete proteins in the four patients evaluable for humoral immune responses. As the generation of an IgG antibody response requires CD4 T cell help for switching antibody class from IgM to IgG, we hypothesize that the development of IgG antibody responses is a surrogate for the generation of a CD4 T cell response against the same antigen. Interestingly, with this personalized tumor vaccine approach, each patient generated an immune response against antigens that were unique to the individual. While an immune response against multiple antigens was induced or boosted, this immune response was still ineffective in controlling the cancer.

We postulate that effective control of cancer will require induction of immunity against a spectrum of cancer antigens, administration of a T cell agonist to boost that immunity and an inhibitor of immune checkpoints to overcome cancer escape mechanisms. We also consider the biggest hurdle for successful immunotherapy is the ability to generate immunity against a spectrum of cancer antigens and have evaluated this autologous DRibble vaccine strategy as an approach to induce that immunity. Many of the IgG antibody responses that developed or were augmented by vaccination were against proteins whose genes were shown by TCGA analyses to be commonly over-expressed in NSCLC, making them possible targets for an anti-cancer immune response.

While the library of peptides that are presented by HLA on the surface of cancer cells is not well studied [43], a recent paper reported that a large number of antibody responses in patients with NSCLC were directed against proteins whose peptides were presented by HLA of NSCLC cell lines [10]. They went on to report that presence of an antibody response correlated with the detection of cytotoxic T cell response against HLA-matched NSCLC cell lines. Consistent with that observation, our group recently reported that the detection of an IgG response following vaccination with a DRibble vaccine, in a preclinical model of breast cancer, correlated with the development of a CD8 T cell response to the same peptide and to the specific mammary cancer cell line [44].

The protoArray data from the current study suggests that CD4 T cells, and hypothetically a coordinated CD8 T cell response, were induced or boosted against a number of proteins whose genes are over-expressed by NSCLC, however, only 1 patient had PBMC and autologous tumor cells available to assess anti-cancer immunity 14 days following their 3rd or 4th vaccination. That this patient developed an increased autologous tumor-specific responses of both type 1 and type 2 cytokines may not be optimal, but it provides insight into modifications to the composition of the vaccine that may further skew strong type 1 cytokines without augmenting type 2 cytokine responses. Additionally, it is thought-provoking that the cytokine response of patient 5 diminished at the time when the patient experienced progression (D85). This fluctuation may have been the result of tumor progression shutting off the immune response, or it may be the result of T cell contraction, a natural component of a T cell response to antigen [45].

We hypothesize that contraction explains the fluctuation in TCR clones we detected in patients receiving an allogeneic DRibble vaccine as adjuvant therapy for NSCLC [46]. This highlights the possibility of combining vaccines with T cell agonists that blunt contraction by augmenting T cell expansion and sustaining antigen-specific T cells [47, 48]. Strategies that monitor expansion and survival of T cell clones may be useful in sorting out the contribution of agents in combination immunotherapy trials which contain cancer vaccines. Development of methods to evaluate contributions of individual agents in combination immunotherapy trials was recently identified as an objective by the US FDA [49]. This is particularly true for the development of T cell agonists, where the receptor is only expressed on the responding T cell for a few days following activation. While preclinical studies have documented that combining cancer vaccines with T cell agonists can increase the therapeutic effect [50–55], their application in the clinic is yet to be reported.

Vaccine strategies for lung cancer, as for most solid tumors, have failed to make a significant impact on survival as monotherapy, even though there is evidence of vaccine-induced anti-cancer immunity [56, 57]. A recent meta-analysis of 18 randomized clinical trials evaluating tumor vaccines and cellular immune therapies in NSCLC reported an average overall survival benefit of 5.4 months (in addition to progression free survival benefit), despite the lack of benefit in the individual studies [58]. The observation that DRibble vaccination was safe and was associated with the development of immune responses against putative cancer-associated antigens in this population of patients with advanced disease is interesting, and would support the concept of combining this strategy with additional immune intervention and evaluating its potential value in earlier stage patients. In addition to checkpoint blockade, costimulatory antibodies have antitumor activity in several models and have significantly augmented therapeutic efficacy of DRibble vaccine in several preclinical models [54, 55].

Given the advanced stage of the patients in this trial, it is not a surprise that a vaccine failed to control tumor growth, and is consistent with the results reported from previous individual vaccine trials in similar patient populations. The ability to assess the value of DRibble vaccination was severely limited by the inability of adequate numbers of patients to complete the entire treatment regimen. Completion of the study was hampered by slow accrual, which was the direct consequence of the generally poor performance status of patients with previously-treated NSCLC who had a malignant pleural effusion suitable for vaccine generation, as well as the time required to make the DRibble preparation in the setting of progressive disease. Evaluation of long-term vaccine administration, disease response, and comparison with immune response was impaired by the rapid clinical decline and short survival of the patient population treated, rendering the investigational plan infeasible. As a next step, our institution is now investigating an allogeneic DRibble vaccine, generated from two established cancer cell lines, in patients with locally-advanced NSCLC.

## Conclusions

Administration of a novel autologous DRibble vaccine in patients with advanced NSCLC was safe in the small population of patients tested. Immune responses against putative cancer-associated antigens were demonstrated in thissmall sample of patients, but further assessment in this study population was infeasible due to their poor prognosis and rapid clinical decline. Further investigation has moved forward with an allogeneic DRibble vaccine for patients with locally-advanced NSCLC.

## Additional files

Additional file 1: Table S1. Patient baseline characteristics. (DOCX 11 kb)

Additional file 2: Figure S1. CONSORT diagram. (DOCX 26 kb)

## Abbreviations

BD: Becton Dickinson
CTCAE: Common Terminology Criteria for Adverse Events
DAMPs: Damage-associated molecular pattern molecules
DRibbles: DRiPs in blebs
DRiPs: Defective ribosomal products
ECOG: Eastern Cooperative Oncology Group
FITC: Fluorescein isothiocyanate
GM-CSF: Granulocyte-Monocyte colony stimulating factor
GPM6A: Glycoprotein M6A
HRP: Horseradish peroxidase
NSCLC: Non-small cell lung cancer
PBMC: Peripheral blood mononuclear cells
PD-1: Programmed cell death receptor-1
RPS6KP1: Ribosomal protein S6 kinase polypeptide 1
RRAGB: Ras-related GTP binding B
SLiPs: Short-lived proteins
