# Supplementary material for: A pilot study of an autologous tumor-derived autophagosome vaccine with docetaxel in patients with stage IV non-small cell lung cancer
Source: J Immunother Cancer. 2017 Dec 19;5:103. doi: 10.1186/s40425-017-0306-6 (PMC5735525; doi:10.1186/s40425-017-0306-6)
Supplement: Supplementary file 1 — Patient baseline characteristics. (DOCX 11 kb) [file 40425_2017_306_MOESM1_ESM.docx]

Additional file 1: Table S1. Patient baseline characteristics

| Patient | 1 | 2 | 3 | 4 | 5 | 6 |
| --- | --- | --- | --- | --- | --- | --- |
| Age | 81 | 54 | 66 | 59 | 63 | 69 |
| Gender | M | F | F | F | M | M |
| Histology | Adeno | Adeno | Adeno | Adeno | Adeno | Adeno |
| Prior chemo | Carbo/Tax | 1.Carbo/Tax  2. Pem | Carbo/Tax/Bev | Cis/Pem | 1.Carbo/Tax/Bev  2. Erlot | Carbo/Tax/Bev |
| Prior radiation | None | Whole Brain | None | 1.Whole Brain  2. Eye | None | None |

Key: M: Male; F: Female; Adeno: Adenocarcinoma; Carbo: Carboplatin; Tax: Paclitaxel; Pem: Pemetrexed; Bev: Bevacizumab; Cis: Cisplatin; Erlot: Erlotinib
