# Supplementary material for: A pilot study of an autologous tumor-derived autophagosome vaccine with docetaxel in patients with stage IV non-small cell lung cancer
Source: J Immunother Cancer. 2017 Dec 19;5:103. doi: 10.1186/s40425-017-0306-6 (PMC5735525; doi:10.1186/s40425-017-0306-6)
Supplement: Supplementary file 2 — CONSORT diagram. (DOCX 26 kb) [file 40425_2017_306_MOESM2_ESM.docx]

Additional file 2: Figure S1. CONSORT diagram
